# Antarctic Krill Oil from *Euphausia superba* Ameliorates Carrageenan-Induced Thrombosis in a Mouse Model

**DOI:** 10.3390/ijms242417440

**Published:** 2023-12-13

**Authors:** Gi Ho Lee, Seung Yeon Lee, Ju Yeon Chae, Jae Won Kim, Jin-Hee Kim, Hye Gwang Jeong

**Affiliations:** 1 Department of Toxicology, College of Pharmacy, Chungnam National University, Daejeon 34134, Republic of Korea; ghk1900@cnu.ac.kr (G.H.L.); sy9842@o.cnu.ac.kr (S.Y.L.); jychae95@o.cnu.ac.kr (J.Y.C.); gjaewon@o.cnu.ac.kr (J.W.K.); 2 Department of Biomedical Laboratory Science, College of Health Science, Cheongju University, Cheongju 28503, Republic of Korea; jinheekim@cju.ac.kr

**Keywords:** krill oil, carrageenan, thrombosis, inflammation, endothelial dysfunction

## Abstract

FJH-KO obtained from Antarctic krill, especially *Euphausia superba*, has been reported to contain high amounts of omega-3 polyunsaturated fatty acids (n-3 PUFA) and to exhibit anticancer and anti-inflammatory properties. However, its antithrombotic effects have not yet been reported. This study aimed to investigate the antithrombotic effects of FJH-KO in carrageenan-induced thrombosis mouse models and human endothelial cells. Thrombosis was induced by carrageenan injection, whereas the mice received FJH-KO pretreatment. FJH-KO attenuated carrageenan-induced thrombus formation in mouse tissue vessels and prolonged tail bleeding. The inhibitory effect of FJH-KO was associated with decreased plasma levels of thromboxane B2, P-selectin, endothelin-1, β-thromboglobulin, platelet factor 4, serotonin, TNF-α, IL-1β, and IL-6. Meanwhile, FJH-KO induced plasma levels of prostacyclin I2 and plasminogen. In vitro, FJH-KO decreased the adhesion of THP-1 monocytes to human endothelial cells stimulated by TNF-α via eNOS activation and NO production. Furthermore, FJH-KO inhibited the expression of TNF-α-induced adhesion molecules such as ICAM-1 and VCAM-1 by suppressing the NF-κB signaling pathway. Taken together, our study demonstrates that FJH-KO protects against carrageenan-induced thrombosis by regulating endothelial cell activation and has potential as an antithrombotic agent.

## 1. Introduction

Cardiovascular disease is closely related to morbidity and mortality in humans worldwide and is the leading cause of reduced quality of life [1]. Thrombosis, a major cause of cardiovascular disease, is characterized by the formation of blood clots inside blood vessels. These blood clots interfere with blood flow and cause various cardiovascular diseases such as myocardial infarction, systemic embolism, stroke, and pulmonary embolism [2,3].

Thrombosis formation begins with vascular injury; in particular, oxidation of lipoproteins and excessive immune responses due to vascular endothelial dysfunction contribute to thrombus formation [4]. Thrombosis is characterized by the formation of blood clots within arteries or veins. Arterial thrombosis is primarily associated with the formation of platelet aggregates on the surface of fissured, ruptured, or ulcerated atherosclerotic plaques, which can eventually cause obstructive or occlusive thrombi, with reduction or interruption in blood flow in the distal arterial territory. Venous thrombosis is mostly associated with the inappropriate activation of the coagulation cascade. Inflammation in the endothelium triggers the activation of the coagulation cascade, consequently exacerbating stasis of blood flow and enhancing the imbalance of coagulation factors towards a prothrombotic state [5]. Endothelial dysfunction reduces the production of nitric oxide (NO), an important biomolecule involved in vascular tone homeostasis. Low levels of NO in blood vessels are associated with impaired vasodilation and enhanced platelet function [6]. Furthermore, endothelial activation facilitates adhesion and infiltration of immune cells by increasing the expression of inflammatory molecules, such as vascular cell adhesion molecule 1 (VCAM-1), intercellular adhesion molecule 1 (ICAM-1), and P-selectin, thus generating an excessive inflammatory response [7,8]. It ultimately plays a central role in the initiation and progression of thrombosis by promoting the adhesion and aggregation of activated platelets and the generation of fibrin at the site of damaged endothelial cells [9,10].

Inflammation is a key factor in thrombosis initiation and progression. The activation of tissue factor (TF) by proinflammatory cytokines such as tumor necrosis factor-α (TNF-α), interleukin (IL)-1β, IL-6, and monocyte chemoattractant protein 1 (MCP-1) leads to the induction of a procoagulant state [11,12]. TF, which is recognized as a prothrombotic mediator, is found in leukocytes and acts as a trigger for inflammation by promoting platelet adhesion and recruitment to damaged endothelial cells and blood vessel walls. Subsequently, thromboxane B2 (TXB2) and adenosine diphosphate secreted from activated platelets induce platelet aggregation and promote thrombus formation [12,13].

Aspirin is one of the antiplatelet agents currently used to prevent and treat thrombosis. In particular, low doses of aspirin, a non-steroidal anti-inflammatory drug (NSAID), are used to treat thrombosis. It inhibits the aggregation of platelets and the formation of blood clots by targeting the enzyme cyclooxygenase. Antithrombotic agents are effective and improve patient prognosis. However, bleeding, as an unwanted side effect, is heightened by liver and kidney dysfunction, increasing this risk [14,15]. Thus, there is an urgent need to identify new agents that can provide potent antithrombotic effects with fewer adverse side effects. In addition, when evaluating the anti-thrombotic effect, aspirin, which possesses both anti-inflammatory and anti-thrombotic effects, is suitable as a comparative drug.

Antarctic krill oil (FJH-KO) is obtained from Antarctic krill, specifically *Euphausia superba*, a small shrimp-like crustacean that serves as a rich source of omega-3 polyunsaturated fatty acids (n-3 PUFA). The fatty acids in FJH-KO are bound to phospholipids, particularly phosphatidylcholine [16,17]. This unique composition has the potential to enhance the bioavailability of n-3 PUFAs in various tissues. Several studies have reported that the consumption of n-3 PUFAs can decrease the platelet-mediated generation of thrombin and reduce factors that contribute to blood clot formation [18,19]. Previous studies have reported that FJH-KO supplementation attenuates the inflammatory response of chondrocytes in an animal model of osteoarthritis [20,21]. However, the antithrombotic effect of FJH-KO has not yet been studied. Therefore, in this study, we investigated the antithrombotic effects of FJH-KO in a carrageenan-induced mouse model and in human endothelial cells.

## 2. Results

### 2.1. FJH-KO Attenuates Carrageenan-Induced Thrombosis in Mouse Tail

**Figure 1:**
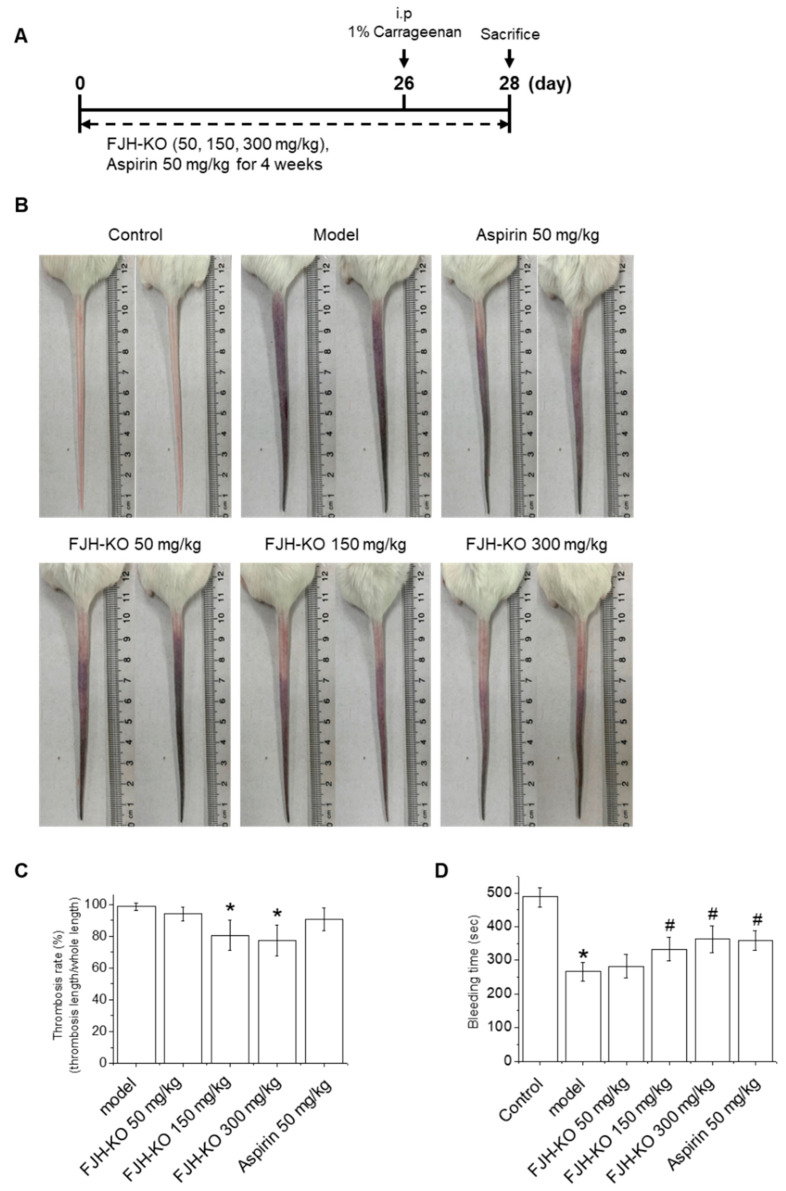
Effects of FJH-KO on carrageenan-induced thrombosis in mice. (**A**) Schematic diagram of carrageenan-induced thrombosis model with FJH-KO and aspirin treatment. (**B**) Thrombus length, (**C**) thrombosis rate (thrombosis length/whole tail length), and (**D**) bleeding time (s) in the tail of carrageenan-induced thrombosis mice. * *p* < 0.05 vs. the vehicle control group, ^#^ *p* < 0.05 vs. the carrageenan model group (n = 8).

To investigate the effects of FJH-KO on thrombosis, ICR mice were subjected to the treatment schedule shown in Figure 1A. Briefly, the mice received daily intragastric administration of vehicle (corn oil), FJH-KO, or aspirin for 4 weeks. On day 26, all mice were administered an intraperitoneal (i.p.) injection of 1% carrageenan solution (50 mg/kg body weight). As shown in Figure 1B, the tail of the mice changed to black from the tip after 1% carrageenan injection, indicating the formation of thrombi within the blood vessels of the tails. The black tail length of each treatment group was determined 48 h after carrageenan administration to calculate the thrombosis rate and the relative thrombus length. Compared with the model group, the 150 and 300 mg/kg FJH-KO groups had a significantly inhibited thrombosis rate in mouse tails, whereas the aspirin group showed no significant difference (Figure 1B,C). In addition, the tail bleeding time of the mice was measured 4 h after 1% carrageenan injection, and the model group showed a significant decrease compared to the vehicle control group. Significant differences were observed in the 150 and 300 mg/kg FJH-KO groups compared to the carrageenan model group, and the results were similar to those of aspirin (Figure 1D).

### 2.2. Effects of FJH-KO on Thrombosis Parameters

**Figure 2:**
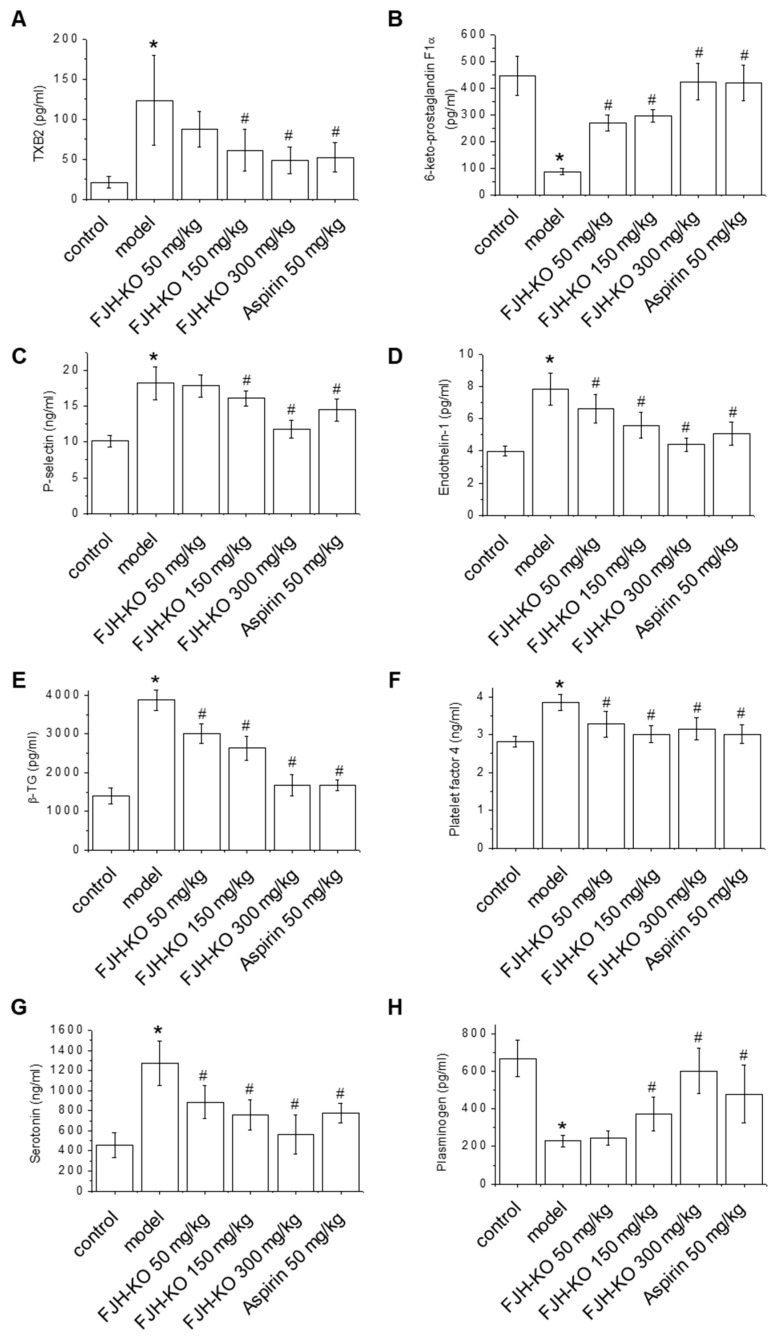
Effect of orally administered FJH-KO on the plasma levels of (**A**) thromboxane B2 (TXB2), (**B**) 6-keto prostaglandin F1α (6-keto PGF1α), (**C**) P-selectin, (**D**) endothelin-1 (ET-1), (**E**) β-thromboglobulin (β-TG), (**F**) platelet factor 4 (PF4), (**G**) serotonin, and (**H**) plasminogen in carrageenan-induced thrombosis model mice. * *p* < 0.05 vs. the vehicle control group, ^#^ *p* < 0.05 vs. the carrageenan model group (n = 8).

Endothelin-1 (ET-1) and TXB2 have a prothrombotic effect, while prostacyclin I2 (PGI2) has a role in anti-platelet aggregation, thus preventing thrombosis [22]. In this study, thrombosis parameters were investigated to evaluate the effects of FJH-KO on thrombus formation. As shown in Figure 2A,B, the level of TXB2 significantly increased, whereas that of 6-keto-prostaglandin F1α (6-keto PGF1α), an inactive metabolite derived from prostaglandin I2 (PGI2), decreased in carrageenan-induced mouse plasma. In contrast, the 150 and 300 FJH-KO mg/kg groups had a significantly reduced plasma level of TXB2, and an increased plasma level of 6-keto PGF1α in a concentration-dependent manner compared with the carrageenan model group (Figure 2A,B). As shown in Figure 2C,D, carrageenan treatment increased the plasma levels of P-selectin and ET-1, indicating vascular endothelial dysfunction. Compared to the carrageenan model group, the FJH-KO and aspirin groups exhibited a dose-dependent decrease in the plasma levels of P-selectin and ET-1, further confirming the antithrombotic effects of FJH-KO (Figure 2C,D).

The plasma levels of β-thromboglobulin (β-TG), platelet factor 4 (PF4), and serotonin were measured to evaluate platelet activation in the carrageenan-induced thrombosis mouse model [23]. Carrageenan treatment induced the plasma levels of β-TG and PF4, whereas FJH-KO treatment effectively attenuated the carrageenan-induced plasma levels of β-TG and PF4, similar to the aspirin group (Figure 2E,F). In addition, the serotonin levels in the carrageenan model group were markedly higher than those in the vehicle control group. However, similar to the aspirin group, the FJH-KO group showed decreased plasma serotonin levels in the carrageenan-treated mouse model (Figure 2G). The FJH-KO group showed a concentration-dependent increase in plasma plasminogen levels in the carrageenan-induced mouse model, consistent with the observation in the positive control aspirin group (Figure 2H).

### 2.3. Effects of FJH-KO on Proinflammatory Cytokine Levels in Mouse Plasma

**Figure 3:**
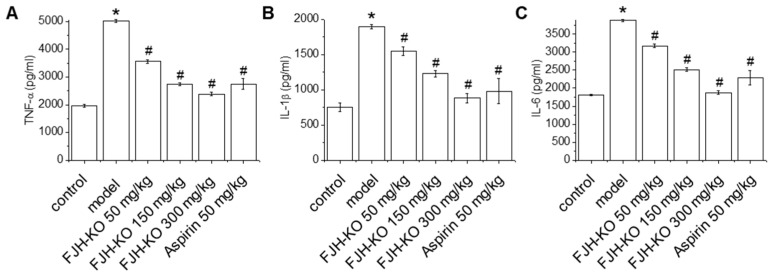
Effect of orally administered FJH-KO on the plasma levels of (**A**) tumor necrosis factor alpha (TNF-α), (**B**) interleukin (IL)-1β, and (**C**) IL-6 in carrageenan-induced thrombosis model mice. * *p* < 0.05 vs. the vehicle control group, ^#^ *p* < 0.05 vs. the carrageenan model group (n = 8).

Proinflammatory cytokines, such as TNF-α, IL-1β, and IL-6, weaken anticoagulant activity, cause vasoconstriction, and promote thrombosis [11,12]. Carrageenan treatment markedly induced the plasma level of TNF-α, IL-1β, and IL-6 compared with the vehicle control group (Figure 3). FJH-KO treatment reduced the carrageenan-induced plasma levels of TNF-α, IL-1β, and IL-6 in a concentration-dependent manner. Similarly, the aspirin group showed decreased plasma TNF-α, IL-1β, and IL-6 levels compared with the carrageenan-induced model group.

### 2.4. Effects of FJH-KO on Vascular Endothelial Dysfunction and Inflammation in Mouse Blood Vessels

**Figure 4:**
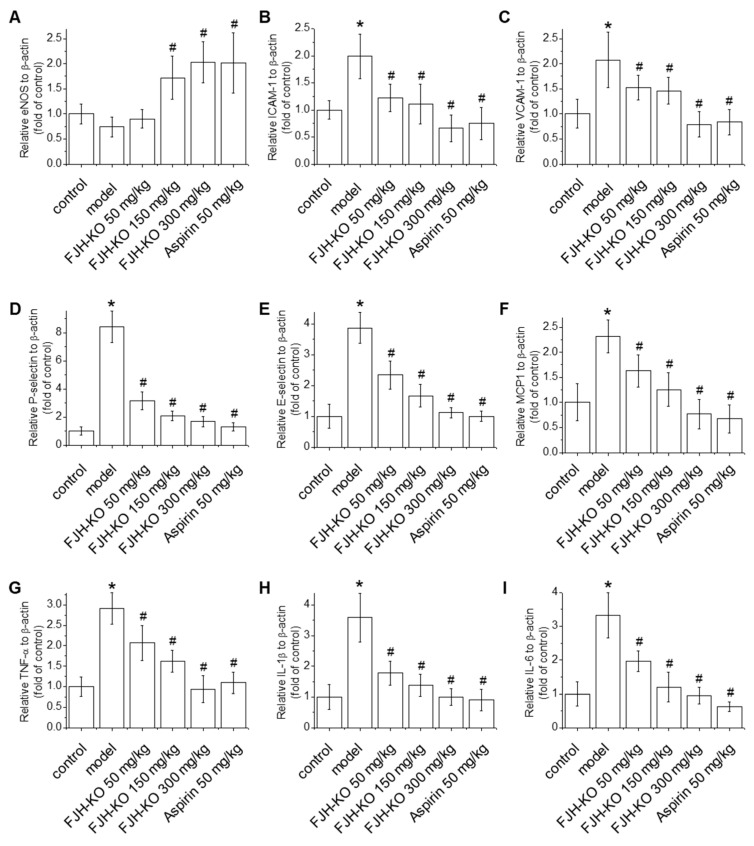
Effect of FJH-KO on the expression of blood clot-related mRNAs in carrageenan-induced mouse blood vessels. (**A**) Endothelial nitric oxide synthase (eNOS), (**B**) intercellular adhesion molecule 1 (ICAM-1), (**C**) vascular cell adhesion molecule 1 (VCAM-1), (**D**) P-selectin, (**E**) E-selectin, (**F**) monocyte chemoattractant protein 1 (MCP-1), (**G**) TNF-α, (**H**) IL-1β, and (**I**) IL-6. * *p* < 0.05 vs. the vehicle control group, ^#^ *p* < 0.05 vs. the carrageenan model group (n = 8).

Endothelial dysfunction leads to increased oxidation of lipoproteins within blood vessels and disrupts immune responses, ultimately leading to thrombus formation [4]. Evaluation of the effect of FJH-KO on endothelial dysfunction revealed that carrageenan treatment reduced the mRNA levels of endothelial nitric oxide synthase (eNOS), although this was not statistically significant. Meanwhile, the 150 mg/kg FJH-KO, 300 mg/kg FJH-KO, and aspirin groups showed markedly increased eNOS mRNA levels compared with the carrageenan group (Figure 4A). In addition, the mRNA levels of VCAM-1, ICAM-1, P-selectin, E-selectin, and MCP-1 were significantly lower in the 50, 150, and 300 FJH-KO mg/kg and aspirin groups than in the carrageenan group (Figure 4B–F). Similarly, carrageenan treatment markedly induced the mRNA levels of TNF-α, IL-1β, and IL-6 compared with the vehicle control group (Figure 4G–I), and these carrageenan-induced increases in mRNA levels were reduced by FJH-KO treatment in a concentration-dependent manner, suggesting that FJH-KO is a potent suppressor of endothelial dysfunction.

### 2.5. Effects of FJH-KO on Carrageenan-Induced Tissue Thrombosis in Mice

**Figure 5:**
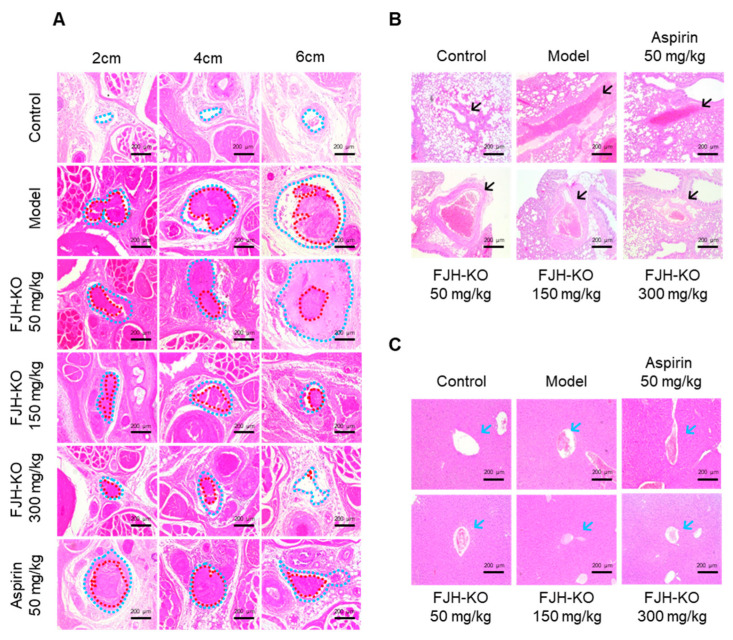
Effects of FJH-KO on carrageenan-induced thrombosis in mouse tissues. (**A**) Hematoxylin and eosin (H&E) staining of the tail 2, 4, and 6 cm from the tail tip (Scale bar: 200 μm). Vessels and thrombi are represented by blue and red dotted lines, respectively. (**B**) H&E staining of lung tissue (Scale bar: 200 μm). Venous thrombosis is represented by black arrows. (**C**) H&E staining of liver tissue (Scale bar: 200 μm). Hepatic vein thrombosis is represented by blue arrows.

The mice were euthanized 48 h after the carrageenan injection, and tissues from the tail, liver, and lungs were collected for hematoxylin and eosin (H&E) analysis. In the carrageenan model group, the tail vessels (blue dotted line) located at the 2 and 4 cm positions were almost completely blocked by a blood clot, as indicated by the red dotted lines. In contrast, approximately 80% of the vessels located at the 6 cm position were also blocked by thrombi (Figure 5A). The FJH-KO group showed inhibition of thrombus formation in a concentration-dependent manner, whereas the aspirin group showed weaker inhibition of thrombus formation at the 6 cm position compared with the FJH-KO group. Compared to the vehicle control group, the lung tissue of the carrageenan model group showed venous thrombosis (black arrows), whereas the FJH-KO groups showed attenuated thrombus formation in a concentration-dependent manner (Figure 5B). The antithrombotic effect was less potent in the aspirin group than in the FJH-KO group. Similarly, the liver tissue of the carrageenan model group showed hepatic vein thrombosis (blue arrows), whereas the FJH-KO group showed attenuated thrombosis formation in a concentration-dependent manner (Figure 5C).

### 2.6. Effects of FJH-KO on NO Production and eNOS Phosphorylation in Endothelial Cells

**Figure 6:**
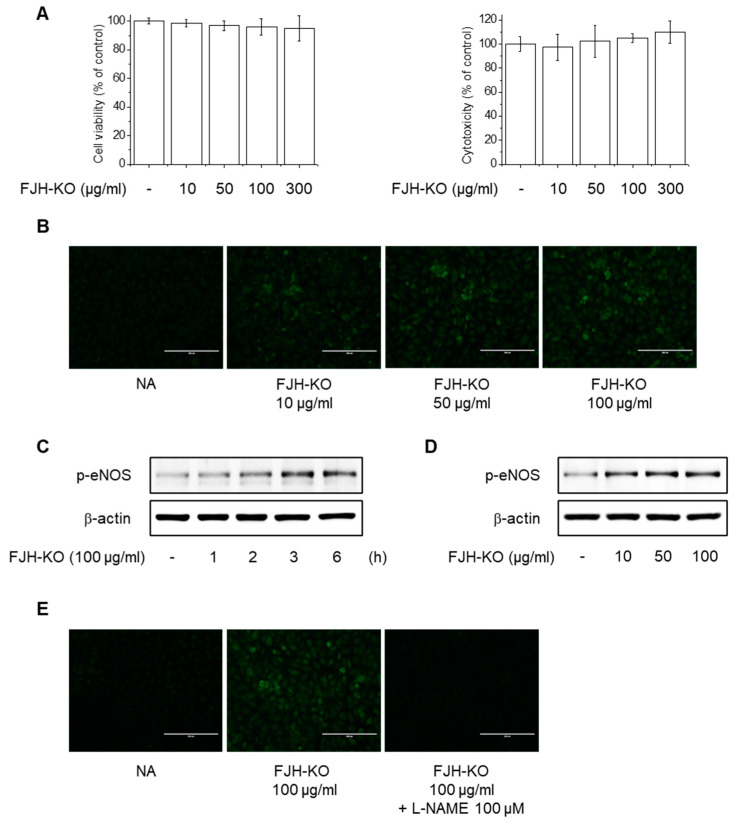
Effect of FJH-KO on eNOS phosphorylation and nitric oxide (NO) production in EA.hy926 cells. (**A**) Cells were treated with 10–300 μg/mL FJH-KO for 24 h. Cell viability and cytotoxicity were determined using MTT and LDH assays, respectively. (**B**) Cells were treated with 10–100 μg/mL FJH-KO for 3 h. NO generation was detected using DAF-2DA (Scale bar: 200 μm). (**C**,**D**) Cells were treated with 100 μg/mL FJH-KO for 1–6 h or 10–100 μg/mL FJH-KO for 3 h and assessed via Western blotting. (**E**) Cells were pretreated with 100 μM L-NAME for 1 h and were then treated with 100 μg/mL FJH-KO for 3 h. NO generation was detected using DAF-2DA (Scale bar: 200 μm).

**Figure 7:**
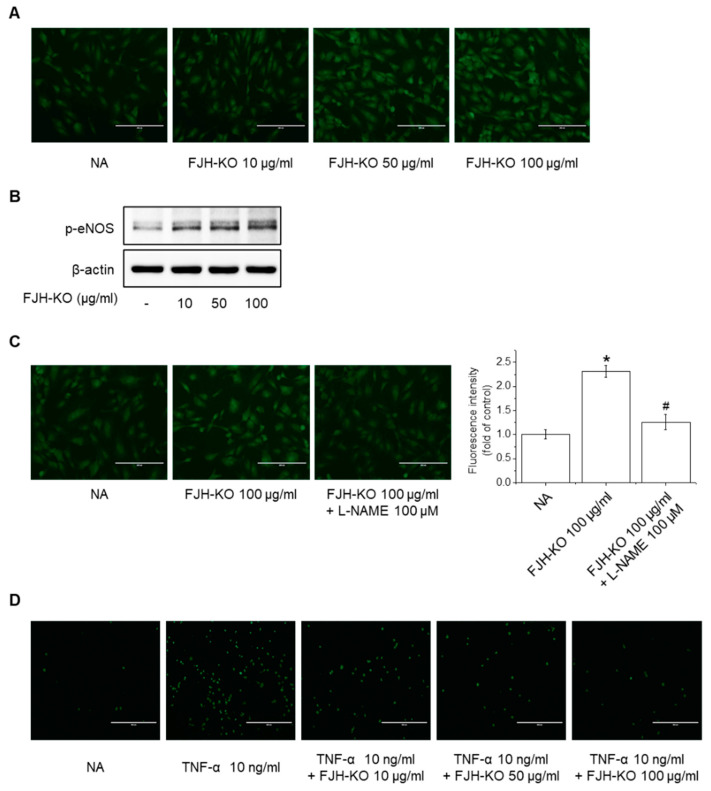
Effect of FJH-KO on NO production and THP-1 monocyte adhesion in HUVEC cells. (**A**) Cells were treated with 10–100 μg/mL FJH-KO for 3 h. NO generation was detected using DAF-2DA (Scale bar: 200 μm). (**B**) Cells were treated with 10–100 μg/mL FJH-KO for 3 h and assessed via Western blotting. (**C**) Cells were pretreated with 100 μM L-NAME for 1 h and were then treated with 100 μg/mL FJH-KO for 3 h. NO generation was detected using DAF-2DA (Scale bar: 200 μm). (**D**) Cells were treated with 10–100 μg/mL of FJH-KO for 3 h, followed by incubation with 10 ng/mL TNF-α for 12 h. Endothelial cells were co-cultured with THP-1 cells for 1 h, and the adherence of endothelial cells to monocytes was assessed via fluorescence microscopy (Scale bar: 400 μm). All experiments were performed in triplicate. Data are represented as the mean ± standard deviation. * *p* < 0.01 vs. control cells, ^#^ *p* < 0.01 vs. FJH-KO-treated cells.

Endothelial dysfunction is characterized by a decrease in NO produced by eNOS in endothelial cells and a decrease in NO sensitivity, which ultimately lead to an imbalance in vascular homeostasis, resulting in thrombosis, inflammation, and vessel wall damage that cause various vascular diseases [8,10]. We investigated the effects of FJH-KO on NO production and eNOS phosphorylation in endothelial cells to reveal the mechanism underlying its antithrombotic effect. At the tested concentrations, FJH-KO did not exhibit significant cytotoxicity in EA.hy926 cells (Figure 6A). In the following experiments, the cells were treated with FJH-KO at concentrations ranging from 10 to 100 μg/mL. FJH-KO significantly increased NO production in endothelial cells (Figure 6B and Figure 7A). In addition, treatment with varying concentrations (10–100 μg/mL) of FJH-KO for 3 h increased eNOS phosphorylation in a concentration-dependent manner, with the 100 μg/mL concentration strongly inducing eNOS phosphorylation (Figure 6C,D and Figure 7B). To determine whether eNOS was required for NO production induction in FJH-KO mice, we used NG-nitro-L-arginine methyl ester (L-NAME; an NOS inhibitor) and found that pretreatment with 100 μM L-NAME decreased the FJH-KO-mediated NO production (Figure 6E and Figure 7C).

### 2.7. Effects of FJH-KO on TNF-α-Induced Monocyte Adhesion and Expression of Adhesion Molecules in Endothelial Cells

**Figure 8:**
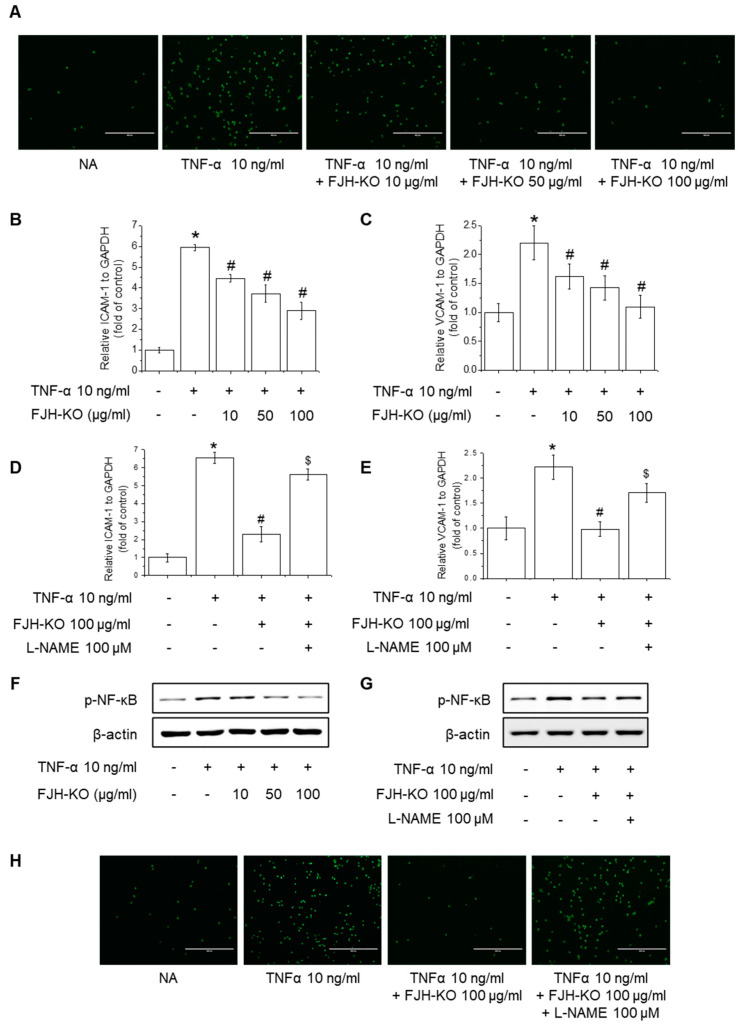
Effects of FJH-KO on TNF-α-induced THP-1 monocyte adhesion in EA.hy926 cells. (**A**) Cells were treated with 10–100 μg/mL FJH-KO for 3 h, followed by incubation with 10 ng/mL TNF-α for 12 h. Endothelial cells were co-cultured with THP-1 cells for 1 h, and the adherence of endothelial cells to monocytes was assessed via fluorescence microscopy (Scale bar: 400 μm). (**B**,**C**) Cells were pretreated with 100 μg/mL FJH-KO for 3 h, followed by 100 ng/mL TNF-α for 12 h. mRNA levels of (**B**) ICAM-1 and (**C**) VCAM-1 were measured using RT-PCR. (**D**,**E**) Cells were pretreated with 100 μM L-NAME for 1 h and then treated with 100 μg/mL FJH-KO for 3 h, followed by incubation with 10 ng/mL TNF-α for 12 h. mRNA levels of (**D**) ICAM-1 and (**E**) VCAM-1 were measured using RT-PCR. (**F**) Cells were pretreated with 10–100 μg/mL FJH-KO for 3 h and were then treated with 10 ng/mL TNF-α for 1 h. (**G**) Cells were pretreated with 100 μM L-NAME for 1 h and were then treated with 100 μg/mL FJH-KO for 3 h, followed by incubation with 10 ng/mL TNF-α for 1 h. NF-κB phosphorylation was assessed using Western blotting. (**H**) Cells were pretreated with 100 μM L-NAME for 1 h and were then treated with 100 μg/mL FJH-KO for 3 h, followed by incubation with 10 ng/mL TNF-α for 12 h. Endothelial cells were co-cultured with THP-1 cells for 1 h, and the adherence of endothelial cells to monocytes was assessed via fluorescence microscopy (Scale bar: 400 μm). All experiments were performed in triplicate. Data are represented as the mean ± standard deviation. * *p* < 0.01 vs. control cells, ^#^ *p* < 0.01 vs. TNF-α-treated cells, and ^$^ *p* < 0.01 vs. FJH-KO-treated cells.

Considering the increasing importance of endothelial dysfunction and leukocyte infiltration in the homeostasis of vascular tone and blood flow, we investigated the effect of FJH-KO on the expression of adhesion molecules and NF-κB signaling induced by TNF-α. Treatment with FJH-KO inhibited the TNF-α-induced adhesion of human monocyte THP-1 cells to endothelial cells (Figure 8A). Moreover, FJH-KO treatment suppressed the TNF-α-induced expression of ICAM-1 and VCAM-1 in a concentration-dependent manner (Figure 8B,C). Notably, treatment with L-NAME significantly attenuated the protective effect of FJH-KO against TNF-α-induced monocyte adhesion, and ICAM-1 and VCAM-1 expression (Figure 8D,E). FJH-KO also demonstrated the suppression of p65 NF-κB phosphorylation, with this inhibitory effect of FJH-KO being reversed by cellular stimulation with L-NAME (Figure 8F,G). In addition, pretreatment with L-NAME attenuated the inhibitory effect of FJH-KO on the TNF-α-induced adhesion of human monocyte THP-1 cells to endothelial cells (Figure 7D and Figure 8H).

## 3. Discussion

In this study, we demonstrate that FJH-KO inhibits carrageenan-induced thrombosis in mice. Our findings indicate that FJH-KO has an antithrombotic effect similar to that of aspirin. In addition, human endothelial cells were used to investigate the underlying mechanisms of FJH-KO, and FJH-KO was found to prevent endothelial dysfunction, which is one of the mechanisms by which FJH-KO prevents thrombosis. To the best of our knowledge, this study is the first to report the antithrombotic effects of FJH-KO.

Carrageenan injection induces thrombus formation within veins, arterioles, and capillaries via intravascular inflammation in mouse tails. In severe instances, this can lead to ischemic necrosis of the mouse tail, which is visually evident through the observation of a blackened tail [16,24]. Therefore, the extent of discoloration in the mouse tail serves as a crucial experimental indicator for intuitively assessing the severity of thrombosis. In this study, FJH-KO and aspirin reduced the black discoloration of mouse tails caused by carrageenan, and FJH-KO mice showed a lower thrombosis rate than aspirin-treated mice. Furthermore, in the evaluation of the effect on tail bleeding time in a carrageenan-induced mouse model, FJH-KO was found to significantly prolong bleeding time, a result similar to that achieved with the commonly used drug aspirin.

In relation to the inhibition of thrombosis, FJH-KO led to a reduction in plasma TXB2 levels while concurrently elevating the plasma levels of 6-keto in the carrageenan-induced mouse model. PGI2 possesses the capacity to inhibit platelet aggregation, whereas TXB2 exerts a contrasting prothrombotic effect [25]. Collectively, these two factors contribute to the regulation of vascular homeostasis and thrombus formation. P-selectin and ET-1 are key proteins that mirror the damage and activation of vascular endothelial cells. Our results showed that FJH-KO reduced the carrageenan-induced plasma levels of P-selectin and ET-1. P-selectin induces monocyte and platelet adhesion to endothelial cells [26], whereas overexpression of ET-1, a potent endogenous vasoconstrictor, triggers disturbances in blood flow [27,28]. Furthermore, FJH-KO effectively attenuated carrageenan-induced plasma levels of β-TG and PF4, which are specific platelet alpha-granule proteins that are released upon platelet activation [29,30] and are measured to evaluate platelet activation in a thrombosis model. Moreover, serotonin released during platelet activation increases aggregation and vessel constriction, thereby playing a major role in hemostasis and thrombus formation [31]. We found that FJH-KO reduced the plasma levels of serotonin in the carrageenan-induced mouse model. Meanwhile, FJH-KO enhanced plasma plasminogen levels, which were decreased by carrageenan. Plasminogen is a precursor protein that can be converted by tissue-type plasminogen activator (tPA) to plasmin, an enzyme playing a crucial role in fibrinolysis. It maintains the normal function of the vascular system by dissolving fibrin, thus contributing to the regulation of blood coagulation [32]. These results suggest that FJH-KO has a protective effect against thrombus formation, similar to aspirin which is currently used as a drug.

Inflammation in blood vessels can cause thrombosis; conversely, thrombi in blood vessels can worsen inflammation [33]. Our results showed that FJH-KO decreased the plasma and mRNA levels of TNF-α, IL-1β, and IL-6 in the carrageenan-induced mouse model. Proinflammatory cytokines exhibit multifaceted effects in thrombosis and blood flow disorders. They impair anticoagulant function by reducing eNOS expression and NO production in endothelial cells while also inducing the expression of vasoconstrictor substances, resulting in vasoconstriction and ultimately promoting thrombosis [6,34]. Therefore, the inhibitory effect of FJH-KO on plasma TNF-α, IL-1β, and IL-6 levels suggests that it regulates inflammation and prevents thrombosis.

Inflammation of the blood vessels generally triggers the activation of endothelial cells, platelets, and leukocytes, leading to endothelial dysfunction and blood clot formation [33,35]. Endothelial dysfunction suppresses eNOS activity and NO production while inducing the expression of adhesion molecules, such as ICAM-1, VCAM-1, and P-selectin, to promote the adhesion of monocytes and platelets to the endothelium. In addition, activated endothelial cells secrete cytokines and chemokines such as TNF-α and MCP-1, which are associated with atherosclerotic plaque formation [36,37]. We found that FJH-KO augmented eNOS expression in carrageenan-stimulated mouse blood vessels and triggered eNOS phosphorylation along with enhanced NO production in human endothelial cells. Moreover, FJH-KO not only decreased the expression of adhesion molecules (ICAM-1, VCAM-1, P-selectin, and E-selectin) and proinflammatory mediators (TNF-α, IL-1β, IL-6, and MCP-1) in carrageenan-induced mouse blood vessels but also inhibited the TNF-α-mediated expression of ICAM-1 and VCAM-1 in human endothelial cells, thereby reducing monocyte adhesion. NF-κB, a key transcription factor in the inflammatory response, activates endothelial cells, leading to an increase in the expression of adhesion molecules and cytokines. Ultimately, this disrupts the balance between coagulation and fibrinolysis, leading to thrombosis [37,38]. Our result showed that FJH-KO attenuated TNF-α-induced NF-κB phosphorylation, whereas pretreatment with the NOS inhibitor L-NAME reversed the effect of FJH-KO on NF-κB phosphorylation and monocyte adhesion in human endothelial cells. Consistent with our findings, n-3 PUFA has been reported to induce eNOS activation and NO-mediated coronary artery relaxation and improve endothelial dysfunction by regulating the imbalance between endothelium-derived factors through inhibition of NF-κB activity [39,40]. Some studies using animal models of arterial or venous thrombosis indicate that supplemental intake of n-3 PUFAs can alter thrombosis [41,42]. These results suggest that the preventive effect of FJH-KO against inflammation and endothelial dysfunction may explain the improved outcomes of FJH-KO in carrageenan-induced thrombosis.

**Figure 9:**
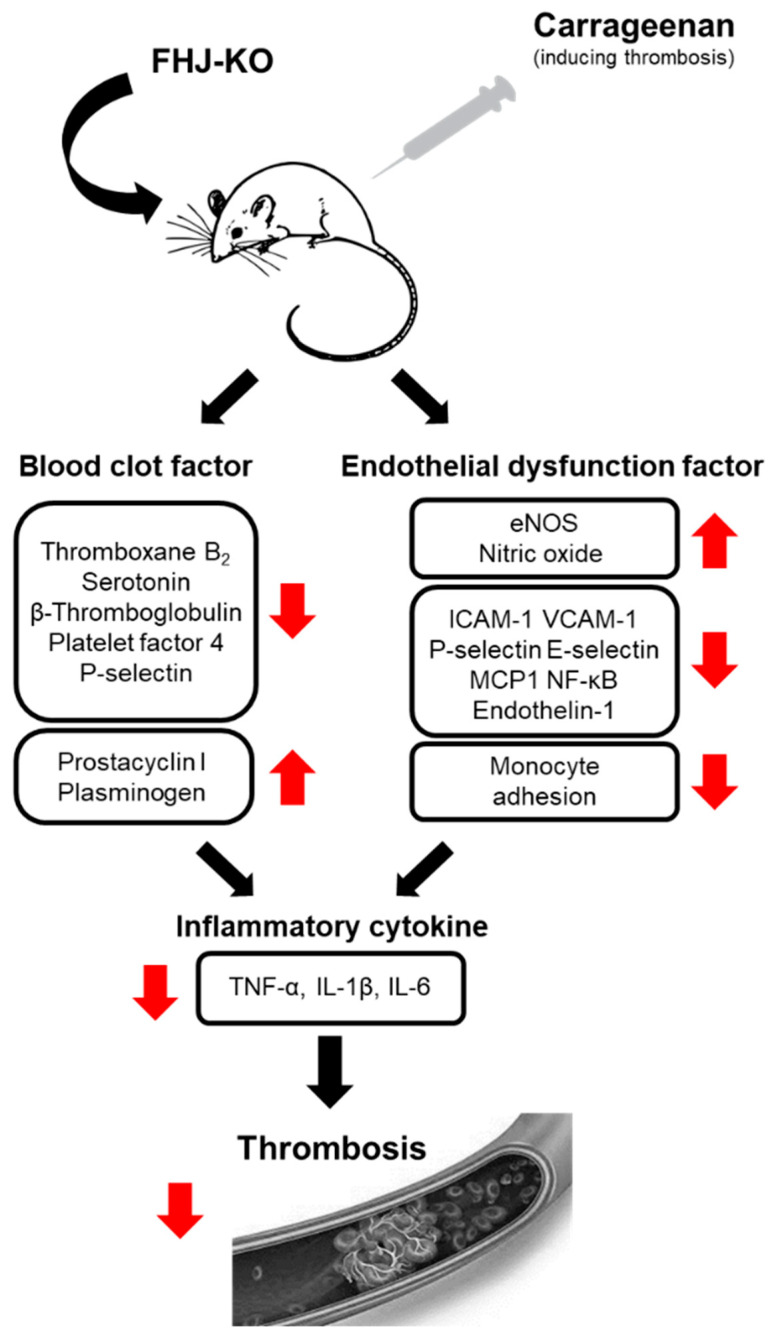
Schematic diagram of the protective effect of FJH-KO on thrombosis by regulating endothelial dysfunction and inflammation.

Taken together, we demonstrated that oral administration of FJH-KO protected against carrageenan-induced thrombosis in mouse tissue vessels by decreasing the expression of adhesion molecules and the secretion of proinflammatory cytokines. We also found antithrombotic functions of FJH-KO in vitro, such as inhibition of adhesion molecules and adhesion of monocytes to endothelial cells. Furthermore, we suggested that FJH-KO attenuates thrombosis, including increased NO production and reduced endothelial dysfunction and inflammation (Figure 9). However, this study has some limitations. It primarily focuses on the effect of FJH-KO on carrageenan-induced vascular endothelial dysfunction and inflammation, lacking an in-depth exploration of the mechanism of action of FJH-KO on platelets. Furthermore, additional investigations are necessary to identify the primary active component(s) responsible for the antithrombotic properties of FJH-KO and to elucidate the underlying mechanisms. Nevertheless, our study suggests the potential of FJH-KO as an adjuvant therapy for preventing thrombosis.

## 4. Materials and Methods

### 4.1. Preparation of the Extract

The Antarctic krill oil (FJH-KO) was supplied by Frombio (Suwon-si, Gyeonggi-do, Republic of Korea). The extracts were stored at −20 °C until use. The nutritional components of FJH-KO were the same as those reported in previous studies, and the sum of eicosapentaenoic acid (EPA) and docosahexaenoic acid (DHA) in FJH-KO was 204.49 ± 2.72 mg/g [20,21].

### 4.2. Chemicals and Reagents

Dulbecco’s modified Eagle’s medium (DMEM), fetal bovine serum (FBS), streptomycin, and penicillin were obtained from Welgene (Gyeongsan, Republic of Korea), and Endothelial Growth Medium 2 (EGM-2) Bullet Kit medium was purchased from Lonza Bioscience (Walkersville, MD, USA). FJH-KO was acquired from Frombio (Suwon-si, Republic of Korea) and κ-Carrageenan, aspirin, and TNF-α were purchased from Sigma-Aldrich (St. Louis, MO, USA). L-NAME was purchased from Calbiochem (San Diego, CA, USA). Enzyme-linked immunosorbent assay (ELISA) kits for TXB2, P-selectin, ET-1, β-TG, platelet factor 4, plasminogen, TNF-α, IL-1β, and IL-6 were acquired from R&D Systems (Minneapolis, MN, USA) and that for 6-keto-prostagladnin F1α was from Cusabio Technology (Houston, TX, USA). Serotonin was from Abcam (Cambridge, MA, USA). 3-(4,5-dimethylthiazol-2-yl)-2,5-diphenyltetrazolium bromide (MTT) was obtained from USB Corporation (Cleveland, OH, USA). A lactate dehydrogenase (LDH) assay kit was purchased from Roche Applied Science (Indianapolis, IN, USA). All kits were used according to the manufacturers’ instructions. 4,5-diaminofluorescein diacetate was obtained from Invitrogen (Waltham, MA, USA). PCR primers were custom-synthesized by Bioneer Co. (Daejeon, Republic of Korea). The primary antibodies against p-eNOS and p-NF-κB and secondary antibodies were purchased from Cell Signaling Technology (Danvers, MA, USA). β-actin was purchased from Santa Cruz Biotechnology (Dallas, TX, USA). All chemicals were of the highest commercially available grade.

### 4.3. Carrageenan-Induced Thrombosis Mouse Model

Six-week-old male ICR mice were purchased from Raon Bio (Yongin, Republic of Korea). The mice were allowed free access to food and water and maintained in a room with controlled environmental conditions (22 ± 2 °C, 50 ± 5%, 12:12 h light/dark cycle). After 1 week of acclimatization, the mice were randomly divided into six groups (n = 8 per group): control, carrageenan, carrageenan + 50 mg/kg FJH-KO, carrageenan + 150 mg/kg FJH-KO, carrageenan + 300 mg/kg FJH-KO, and carrageenan + 50 mg/kg aspirin. All mice except those in the vehicle control group were injected i.p. with 100 μL of 1% carrageenan solution (equivalent to 50 mg/kg body weight) on day 26. Two days after carrageenan injection, all mice were anesthetized in a CO_2_ chamber and their tails were imaged immediately after. The blood, tail, lung, liver, and blood vessel samples were collected separately. The experimental mouse protocol was approved by the Committee on Ethics of Animal Experiments of Chungnam National University (202212A-CNU-244).

### 4.4. Tail Bleeding Assay

A 1 cm segment of the tail was amputated and submerged in 50 mL of saline prewarmed in a water bath at 37 °C, with the tail in a vertical position. The bleeding time was determined by measuring with a stopclock for up to 20 min. Each animal was monitored for 10 min to detect rebleeding, even if bleeding ceased. If rebleeding occurred, the sum of the bleeding times within 20 min was used. The experiment was terminated after 20 min to avoid lethality as required by the local animal ethics committee.

### 4.5. Thrombus Length Measurement

The length of the thrombus and the full length of the tail were measured using a ruler. The thrombosis rate of the mouse tail was calculated using the following formula: thrombus length/whole tail length × 100.

### 4.6. Cell Culture and Treatment

Human endothelial cell line EA.hy926 was obtained from the American Type Culture Collection (Bethesda, MD, USA) and maintained in high-glucose DMEM supplemented with 10% heat-inactivated FBS and penicillin–streptomycin. Human umbilical vein endothelial cells (HUVECs) were purchased from Lonza Bioscience and cultured in EGM-2 Bullet Kit medium containing growth supplements. The cells were incubated at 37 °C in an incubator containing 5% CO_2_. HUVECs at passages 5–7 were used. Upon reaching 85~90% confluency, the cells were treated with FJH-KO at varying concentrations (10, 50, and 100 μg/mL), L-NAME (100 μM), and TNF-α (10 ng/mL).

### 4.7. Cell Viability and Cytotoxicity Assay

Cell viability and cytotoxicity were assessed using MTT and LDH assays, respectively, to determine the toxicity of FJH-KO to EA.hy926 cells. The cells (1 × 10^5^ cells/well) were seeded in 96-well plates with DMEM containing 10% FBS and incubated for 24 h. The following day, varying concentrations of FJH-KO (10–300 μg/mL) were added and incubated for 24 h. MTT solution was added at 37 °C and 5% CO_2_ for 30 min, after which the medium was discarded and formazan crystals were solubilized with dimethylsulfoxide. Absorbance was measured at 550 nm using a BioTek Synergy HT microplate reader (BioTek Instruments, Winooski, VT, USA). The medium was collected for the LDH assay and mixed with LDH solution. Absorbance was measured at 490 nm using a microplate reader.

### 4.8. Real-Time PCR

Total RNA was isolated from blood vessel tissues and endothelial cells using RNAiso Plus (Total RNA extraction reagent; Takara Bio Inc., Shiga, Japan) and reverse transcribed to cDNA using the BioFact RT Series kit (BioFact, Daejeon, Republic of Korea). PCR amplification was monitored using the Bio-Rad CFX Connect Real-Time PCR software (version 1.4.1; Bio-Rad Laboratories, Hercules, CA, USA). The primer pairs used are listed in Table 1.

### 4.9. Western Blotting

Cells were lysed in CETi lysis buffer (120 mM NaCl, 40 mM Tris [pH 8], and 0.1% Nonidet P-40; TransLab, Daejeon, Republic of Korea) on ice for 30 min and centrifuged at 13,000 rpm for 15 min. The supernatant was collected as the source of the sample protein, and the concentration was determined at 595 nm using a protein assay kit (PRO-MEASURE™ Protein Measurement Solution; Intron Biotechnology, Seongnam, Republic of Korea). Equal amounts of total cellular protein were separated by 10% sodium dodecyl sulfate-polyacrylamide gel electrophoresis and electroblotted onto polyvinylidene difluoride membranes. The membranes were blocked with 5% skim milk for 1 h. Primary antibodies were added overnight, followed by secondary anti-mouse or anti-rabbit antibodies, as appropriate. Protein bands were visualized using an Enhanced Hisol ECL Plus Western Blot Detection Kit (BioFact).

### 4.10. Hematoxylin and Eosin (H&E) Staining

After the experiments were completed, tissues from the liver, lungs, and tail were extracted. The tail segment was amputated at 2, 4, and 6 cm. All tissues were fixed in 10% formalin for 48 h, made transparent in xylene, and processed into paraffin blocks. The manufactured block was cut into 3–4 μm thick sections using a microtome, deparaffinized and hydrated, and then observed under an optical microscope through H&E staining to distinguish the structural characteristics of tissues.

### 4.11. ELISA

Plasma concentrations were assessed using ELISA kits according to the manufacturer’s instructions. The wells of the plate were coated with specific antibodies, and control, test, and standard samples were added to the wells. Biotinylated-specific antibodies were incubated with standards and samples at room temperature. Horseradish peroxidase-conjugated streptavidin was added, after which the wells were washed and 3,3’,5,5’-tetramethylbenzidine solution was added. Finally, the absorbance of the stop solution was measured at 450 nm using a spectrophotometer. Concentrations were determined using standard plot graphs.

### 4.12. Measurement of NO Production

NO production was visualized and quantified using the NO-specific fluorescent dye DAF-2DA. Cells were cultured in 48-well plates (1 × 10^5^ cells/well) and loaded with DAF-2DA at a final concentration of 5 μM for 30 min at 37 °C, rinsed three times with HBSS to remove the excess probe, and kept in the dark. The cells were then treated with various concentrations of FJH-KO. The cells were fixed in 5% paraformaldehyde for 5 min and viewed under an EVOS fluorescence microscope (Life Technologies, Carlsbad, CA, USA).

### 4.13. Monocyte Adhesion

Cells were seeded in 48-well plates (1 × 10^5^ cells/well) and were preincubated with FJH-KO for 3 h followed by 10 ng/mL TNF-α for 12 h. THP-1 cells were fluorescentlylabeled using calcein-AM (100 μM, 1 × 10^6^ cells), and the cells were then incubated for 1 h. The wells were washed twice with phosphate-buffered saline to remove nonadherent cells. The cells were imaged using an EVOS fluorescence microscope.

### 4.14. Statistical Analysis

The experiments were performed at least three times, and the results are expressed as the mean ± standard deviation (SD). The animal study data are shown as the mean ± SD (n = 8). Statistical significance was evaluated using a one-way analysis of variance followed by the Tukey–Kramer test. Statistical significance was set at *p* < 0.05 (in vivo) or *p* < 0.01 (in vitro).

## Figures and Tables

**Table 1:** The primer sequence used for real-time PCR.

Gene	Sequences	
*Mouse β-actin*	F	AGCCATGTACGTAGCCATCC
	R	CTCTCAGCTGTGGTGGTGAA
*Mouse eNOS*	F	CGCAAGAGGAAGGAGTCTAGCA
	R	TCGAGCAAAGGCACAGAAGTGG
*Mouse ICAM-1*	F	AAACCAGACCCTGGAACTGCAC
	R	GCCTGGCATTTCAGAGTCTGCT
*Mouse VCAM-1*	F	GCTATGAGGATGGAAGACTCTGG
	R	ACTTGTGCAGCCACCTGAGATC
*Mouse P-Selectin*	F	AAGATGCCTGGCTACTGGACAC
	R	CAAGAGGCTGAACGCAGGTCAT
*Mouse E-Selectin*	F	GGACACCACAAATCCCAGTCTG
	R	TCGCAGGAGAACTCACAACTGG
*Mouse MCP1*	F	GCTACAAGAGGATCACCAGCAG
	R	GTCTGGACCCATTCCTTCTTGG
*Mouse TNF-α*	F	GGTGCCTATGTCTCAGCCTCTT
	R	GCCATAGAACTGATGAGAGGGAG
*Mouse IL-1β*	F	TGGACCTTCCAGGATGAGGACA
	R	GTTCATCTCGGAGCCTGTAGTG
*Mouse IL-6*	F	TACCACTTCACAAGTCGGAGGC
	R	CTGCAAGTGCATCATCGTTGTTC
*Human GAPDH*	F	GGGGAGCCAAAAGGGTCATC
	R	TGGTTCACACCCATGACGAA
*Human ICAM-1*	F	GGCAGCGTAGGGTAAGGTT
	R	CCGGAAGGTGTATGAACTGA
*Human VCAM-1*	F	CAGGCTGTGACTCCCCATTT
	R	CCCTCATTCGTCACCTTCCC

## Data Availability

The data presented in this study are available on request from the corresponding author.
